# Considerations on UAS-Based In Situ Weather Sensing in Winter Precipitation Environments

**DOI:** 10.3390/s25030790

**Published:** 2025-01-28

**Authors:** Gustavo Britto Hupsel de Azevedo, Alyssa Avery, David Schvartzman, Scott Landolt, Stephanie DiVito, Braydon Revard, Jamey D. Jacob

**Affiliations:** 1 School of Mechanical and Aerospace Engineering, Oklahoma State University, Stillwater, OK 74078, USA; 2 Advanced Radar Research Center, University of Oklahoma, Norman, OK 73019, USA; 3 School of Meteorology, University of Oklahoma, Norman, OK 73072, USA; 4 Research Applications Laboratory, National Center for Atmospheric Research, Boulder, CO 80307, USA; 5 Federal Aviation Administration, Washington, DC 20591, USA

**Keywords:** UAV, UAS, WxUAS, weather, winter, precipitation, sensor, in situ

## Abstract

Freezing rain and freezing drizzle can produce nearly undetectable hazards, with potentially catastrophic consequences for aircraft within low altitudes (e.g., the terminal area). However, the lack of direct observations of the low-altitude freezing precipitation environment creates a challenge for forecasters, flight crews, dispatchers, and air traffic controllers. This research demonstrates how unmanned aerial vehicles (UAVs) can be designed and instrumented to create unmanned aerial weather measurement systems (WxUAS) capable of characterizing the low-altitude freezing precipitation environment and providing insight into the mechanisms that govern it. In this article, we discuss the design considerations for WxUAS-based in situ sampling during active precipitation. We present results from controlled experiments at the Oklahoma Mesonet’s calibration laboratory as well as results from intercomparison studies with collocated well-established ground-based instruments in Oklahoma and Colorado. Additionally, we explore the insights provided by high-resolution thermodynamic and cloud droplet size distribution profiles and their potential contributions to a better understanding of the low-altitude freezing precipitation environment.

## 1. Introduction

A considerable portion of the negative economic impacts associated with freezing precipitation is produced by shallow weakly-forced events within the atmosphere’s first 1.5 km [1]. Due to the proximity to the ground and varying range of hydrometeor sizes, some of these precipitation phenomena are not well captured by current weather observation systems (e.g., radar, surface observation stations, and radiosondes), leading to the underrepresentation of freezing precipitation in numerical weather prediction models used in forecasting [2]. From this point forward, numerical weather prediction models used in forecasting are referred to as forecast models.

In aviation, unpredicted and undetected low-altitude freezing precipitation can produce almost invisible hazards, with potentially catastrophic consequences [3]. For this reason, in 2015 the Federal Aviation Administration (FAA) introduced a new rule restricting flights into and out of commercial airports during freezing precipitation events to prevent icing-related accidents. Nonetheless, compliance with this rule requires precise observation and forecasting of precipitation types in the lower troposphere.

In recent years, new tools have been created to integrate data streams from multiple radars, surface and upper air observations, satellites, and forecast models. Some examples of this type of tool include the HEMS (Helicopter Emergency Medical Services) tool by the National Weather Service, the TAIWIN (Terminal Area Icing Weather Information for the Next-Generation Air Transportation System) capability by the National Center for Atmospheric Research and the FAA, and the MRMS (Multi-Radar Multi-Sensor) tool by the National Severe Storms Laboratory. However, coverage, resolution, and detection limitations within current weather observation systems hinder the accurate representation of low-altitude mixed-phased or freezing precipitation in these tools, particularly for freezing rain and freezing drizzle [1,2,4,5,6,7,8].

Additionally, the lack of direct observations of low-altitude freezing precipitation forces ground and space-based observation systems to rely on inferences or numerically generated atmospheric data to produce their hydrometeor classification outputs [2,9,10]. This dependence reduces the operational reliability of the aforementioned data integration tools, leading forecasters, flight crews, dispatchers, and air traffic controllers to rely on human observations (e.g., professional weather observers and pilot reports). Therefore, there is a need for additional low-altitude freezing precipitation observations to inform and train low-altitude forecast tools. Furthermore, the imminent introduction of small delivery Unmanned Aerial Vehicles (UAVs) and Advanced Air Mobility (AAM) transport vehicles into the National Airspace System (NAS) creates a need for low-altitude freezing precipitation observations with high spatiotemporal resolution.

As an initial step towards addressing this critical need, this article presents a new unmanned aerial weather measurement system (WxUAS) designed to sample the low-altitude freezing precipitation environment. The WxUAS prototype provides in situ samples of pressure, temperature, relative humidity, and cloud droplet size distribution. It also offers remote samples of hydrometeor reflectivity and Doppler velocity from an onboard vertically pointing millimeter wave (mmWave) radar. Through these measurements, the WxUAS characterizes the lower troposphere’s thermodynamic structure and microphysical properties. Leveraging the mobility of UAVs, the WxUAS creates a vertical distribution of the measured atmospheric parameters. Repeating these vertical measurements at regular intervals, the WxUAS captures the temporal evolution of the measured parameters’ spatial distribution. These new spatiotemporal measurements within the atmosphere’s lowest 1.5 km can be used for targeted atmospheric studies and in situ validation for new models and hydrometeor classification algorithms. These spatiotemporal measurements also have the potential to be scaled and used to bridge the measurements of the current observation systems, increasing the safety of manned and unmanned flight operations.

Recognizing the complexity of designing and developing a multi-sensor WxUAS for low-altitude winter precipitation measurements, this research and its reporting have been divided into in situ and remote sampling methods. This division enables the reader to better comprehend the intricate details of the measurement techniques in each sampling method. Therefore, this article concentrates on the in situ sampling method within the proposed WxUAS, reserving the details on remote sampling techniques for a future publication. This article presents the design considerations for WxUAS-based in situ sampling in active precipitation (Section 3, Section 4 and Section 5), results from controlled chamber and operational field experiments (Section 6), and the preliminary findings from a small intercomparison study with collocated well-established ground-based instruments during a winter weather event in Colorado (Section 6.3). Although reserved for later publication, some data from the previously mentioned WxUAS-based mmWave radar are presented in this article as corroboration of active precipitation and validation of experimental conditions. Additionally, we explore the insights provided by high-resolution thermodynamic and cloud droplet size distribution profiles and their potential contributions to a better understanding of low-altitude freezing precipitation environments.

## 2. Design Motivation

In near 0 °C environments, radar reflectivity distributions of rain, snow, freezing rain, ice pellets, drizzle, and freezing drizzle can span a 30 dB range and produce overlapping results across precipitation types and intensity [2]. Therefore, the atmosphere’s thermodynamic profile is critical for hydrometeor classification and hazard assessments [11,12]. The current operational observation system for this information is the radiosonde network. However, radiosonde soundings are only available twice daily, resulting in a temporal observation gap larger than the temporal scales of many winter precipitation events.

In addition to low temporal resolution, radiosondes have a sensor exposure issue that hinders their use in winter precipitation studies [1,13]. Due to weight considerations and to their disposable nature, radiosondes deploy fully exposed sensors and leverage airflow induced by the system’s ascending motion through the air. This design produces satisfactory measurements in most environmental conditions; however, during freezing precipitation events, radiosonde sensors can be struck by supercooled drops, resulting in encapsulation in ice. This encapsulation produces errors in portions of radiosonde-based atmospheric profiles [1,13]. Combined with the radiosonde network’s low temporal resolution, this potential freezing bias leads radar-based hydrometeor classification model developers to rely on numerically generated soundings and radar data inferences [2,9].

In light of the aforementioned considerations, this research endeavors to develop and validate an innovative WxUAS in situ sampling technique for measurements in the low-altitude winter precipitation environment as part of a broader WxUAS solution to provide insight into the atmospheric mechanisms that govern it. However, designing WxUAS measurements is challenging because the aircraft can introduce layer mixing (particularly in multirotors), and its components (e.g., motors, batteries, and propeller tips) can introduce heat to the collected samples [14]. Given that precipitation phase can be affected by temperature variations as small as 0.5 °C, meticulous attention to sensor shielding, placement, and sample conditioning are critical for measurement accuracy [14,15,16,17].

To account for these requirements, a WxUAS sensor shield design must be able to expose the sensors to atmospheric conditions (e.g., temperature, moisture, and cloud droplets) without exposing them to precipitation hydrometeors. Although designs in other atmospheric measurement systems account for precipitation, they do not account for WxUAS-specific challenges (e.g., varying tilt angles and relative position). Therefore, this research presents a design proposal that leverages gravity to mechanically separate large precipitation droplets from the air while accounting for other WxUAS-specific challenges (Section 3).

## 3. Sensor Shield

As previously stated, this research aims to provide insight into atmospheric mechanisms that govern the low-altitude winter precipitation environment, particularly in mixed-phased precipitation events that lead to ambiguity and uncertainty in forecast and hazard assessment products. To attain this goal, the proposed WxUAS focuses on temperature, relative humidity, and cloud droplet size distribution. These variables were selected because they can directly reduce ambiguity in other remote measurements, provide an understanding of a large body of weather phenomena, and have SWaP characteristics in accordance with UAS limitations.

In the winter precipitation environment, high-resolution vertical temperature measurements enable the understanding of how temperature changes with height impact precipitation phase as it falls. More specifically, temperature profiles provide information on the height and depth of freezing and melting layers, resolving the precipitation phase observed at the surface. Relative humidity profiles indicate the amount of moisture the atmosphere can absorb before becoming saturated. Additionally, repeated relative humidity profiles allow for an understanding of how certain atmospheric transport mechanisms change the atmospheric structure over time. Finally, the vertical changes in cloud droplet distributions (i.e., amounts and sizes) provide information about visibility conditions for low-level flights and indicate precipitation generation layers that form below radar coverage. The combined spatiotemporal evolution of these parameters helps to elucidate the hazards produced by winter storms and their expected impacts on the surface.

**Figure 1:**
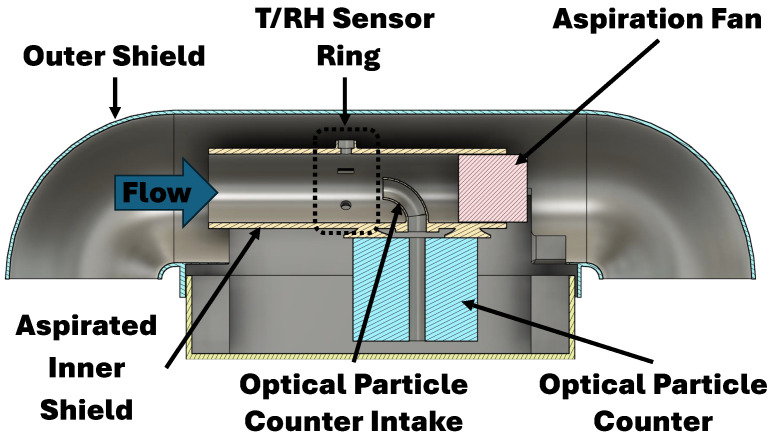
Lateral cross-section of the two-layer custom sensor shield designed to prevent contact with freezing precipitation while allowing for direct atmospheric observation.

**Figure 2:**
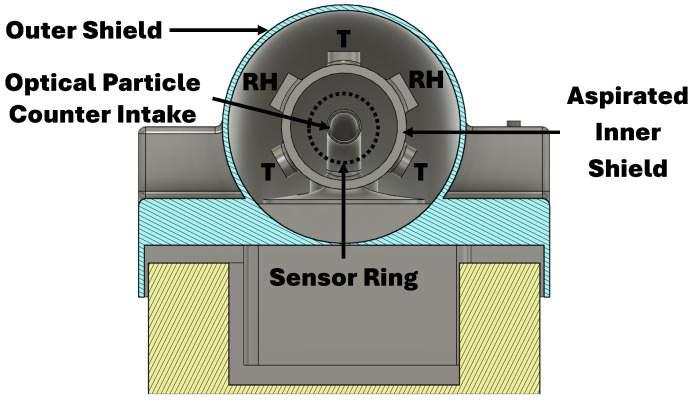
Frontal cross-section view of the two-layer custom sensor shield designed to prevent contact with freezing precipitation while allowing for direct atmospheric observation.

The sensors for these measurements are susceptible to external environmental interference (e.g., sunlight) and require careful sample conditioning for accurate and precise measurements. To reduce the impact of measurement interference sources, the proposed sensor shield employs a cylindrical two-layer solution that optimizes flow conditions over the sensors (Figure 1 and Figure 2).

**Figure 3:**
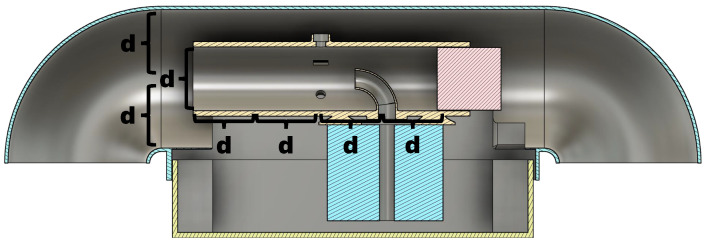
Lateral cross-section view of the two-layer custom sensor shield design, highlighting construction dimensions that enhance flow characteristics, where “d” is the diameter of the inner shield.

The proposed sensor shield shelters three bead thermistors, two capacitive hygrometers, and one optical particle counter. Leveraging the concentric cylindrical solution, the sensors are symmetrically placed around the inner shield’s perimeter equidistant from the center point of a constant flow (greater than 5 ms^−1^). This constant flow is produced by a dedicated aspiration fan. The sensor ring is strategically placed at two-diameter lengths from the aspiration fan and the inner shield’s intake, producing favorable sampling conditions (see Figure 3).

The outer shield is a passive protection layer that guards the inner shield from direct contact with precipitation and sunlight even through the many angle variations that occur during a WxUAS flight. This is possible because the outer shield’s intake extends over the inner shield’s intake and curves downward. As mentioned, only the inner shield is aspirated. This design feature lessens the degree of turbulent heating in the outer shield. Additionally, the outer shield’s diameter is twice as large as the inner shield’s diameter. The resultant air gap insulates the sensor ring from radiative and sensible heat fluxes and impinging water and snow.

Due to its shape, this design can leverage gravity to mechanically separate precipitation from the air. This separation occurs because larger droplets are dominated by their inertia, while small droplets will follow the streamlines on the intake system according to Stokes flow. This design premise is based on the theory of drop trajectory and collection efficiency [18,19]. This flow characteristic allows the system to sample the atmospheric conditions without exposure to harmful freezing precipitation and other external interference sources.

The aspiration rate and interior sensor shield’s diameter produce a flow with an approximate Reynolds number of 10,000. This Reynolds number and the two-diameter distance between the sensor ring and the inner shield’s intake ensure that the sensors have a consistent flow profile, due to a well-mixed flow that is sampled beyond the entrance length (see Figure 3). The Weber numbers for the objective particles at the flow rate range from 3 to 7. These low spectrum numbers denote the minimal presence of droplet smearing and separation within the inner shield.

The above-mentioned flow characteristics assume a shield placement on the vehicle’s topside, away from vehicle-generated interference sources. At this point, vehicle-specific characterization is not discussed because this is a cross-platform solution. This solution’s portability is possible because the sensors are directly integrated into a multiplatform flight control software framework, and the two-layered solution covers various flight angles and relative intake flows. The details of this software integration are among the topics covered in Section 5. Additionally, vehicle-based interference issues have already been discussed in the literature within the scope of each specific measurement [15,20,21,22,23].

## 4. Sensors and Sampling Techniques

**Figure A1:**
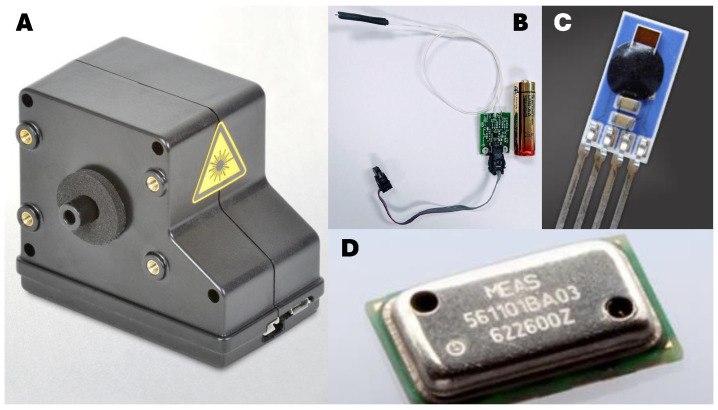
Sensors for in situ measurements: (**A**) OPC-N3 for particle size distribution, (**B**) PT-100 bead thermistor and ADS1115 ADC for fast temperature; (**C**) HYT 271 for slow temperature and relative humidity; and (**D**) MS5611 for fast pressure measurements.

As depicted in Figure 2, the proposed sensor shield houses one optical particle counter (OPC) along with three temperature and two relative humidity sensors. The OPC is the N3 model, manufactured by AlphaSense, Great Notley, GBR. The temperature sensors are Platinum-based bead thermistors with a 100 Ω resistance at 0 °C (PT-100) combined with a 16-bit ADS1115 ADC. The bead thermistor is manufactured by multiple companies while the ADC is produced by Texas Instruments, Dallas, TX, USA. The bead thermistor and ADC are combined and sold as a temperature sensor by InterMet Systems (iMet). The relative humidity sensors are HYT 271 modules manufactured by Innovative Sensing Technologies, Ebnat-Kappel, Switzerland. In addition to these sensors, the WxUAS’s flight controller has a built-in MS5611 digital barometer from TE Connectivity’s Measurement Specialties Sensors, Fremont, CA, USA. An illustration and the sensor specifications are presented in Figure A1 and Table 1.

These sensors were selected based on their quality, resiliency, and low SWaP characteristics. Additionally, the aforementioned pressure, temperature, and relative humidity sensors are widely adopted across the WxUAS research community, and are present in a considerable number of commercial radiosonde solutions. The ubiquity of these sensors is important because it creates a baseline for measurement comparability between different WxUAS and radiosondes, enabling the study of WxUAS design and measurement quality.

**Figure 4:**
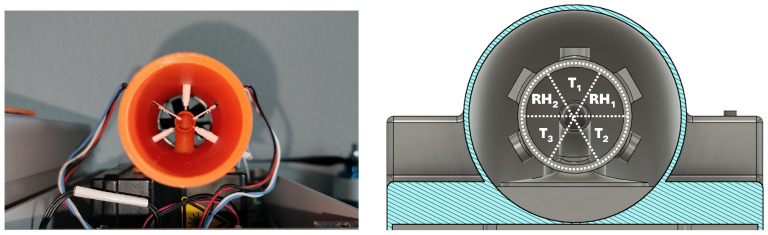
Concentric sensor ring with redundancy for increased accuracy. The right panel illustrates 60° segmentation strategy and the left panel illustrates its implementation.

Leveraging the sensor shield’s cylindrical design (Figure 2) and a sensor redundancy strategy, a concentric sensor ring was created to increase the system’s accuracy. As illustrated in Figure 4, the inner shield’s 360° is divided into six 60° slices. The five temperature and relative humidity sensors occupy the upper slices, while the OPC intake fills the lower slice. Moreover, this innovative concentric design ensures that all sensors experience the same airflow conditions, which enables the evaluation of flow quality metrics and the implementation of measurement quality control and failure-and-recovery algorithms.

The temperature and relative humidity sensors inside the inner shield sample the atmosphere at 10 Hz, while the optical particle counter produces a distribution every second. The higher sampling rate of each thermistor and hygrometer allows for intra-sensor averaging, where every ten samples within a second are averaged to produce higher quality samples at 1 Hz. This technique adds one digit of precision for each sensor, reducing noise and the effects of analog to digital quantization. A similar process is already implemented by the manufacturer on the OPC measurements to produce its 1 Hz data.

Following the intra-sensor averaging process, the temperature measurements from the individual thermistors are averaged to produce a single temperature measurement for the system. A similar multi-sensor averaging procedure is applied to the relative humidity sensors. However, before these system measurements are produced, the sensors’ spatial distribution inside the inner shield is leveraged to evaluate sensor health and intake airflow quality. In these evaluations, each sensor is assessed individually and in comparison to neighboring sensors.

This approach facilitates identifying and removing occasional sampling errors from the final multi-sensor system measurement. Additionally, persistent temporal bias in any individual sensor (e.g., drift) can also be identified, prompting a recalibration or replacement procedure. The neighboring sensor analysis validates the uniform flow assumptions that enable the multi-sensor average. Persistent regional bias detected through this analysis also triggers maintenance procedures. Supplementary system health investigations can be performed using the auxiliary temperature measurements available from the relative humidity sensors. This auxiliary method uses all five upper 60° portions of the inner shield, forming a higher resolution neighboring sensor analysis.

As an example of this enhanced neighboring sensor analysis, consider a profile flight to 200 m above ground level (AGL). In this flight, the ascent rate was 2.5 m/$s^{-1}$, which produces at least 80 measurements after the 1 Hz intra-sensor averaging. For the sake of conciseness, consider four moments to represent varied flight conditions that can produce airflow disturbances: take-off acceleration (at 2 m), developed flight 1 (at 70 m), developed flight 2 (at 130 m), and profile top deceleration (at 192 m). The choice to represent two developed flight stages accounts for potential wind gusts that can disturb the constant ascent assumptions and produce sharp changes in aircraft angle.

**Figure 5:**
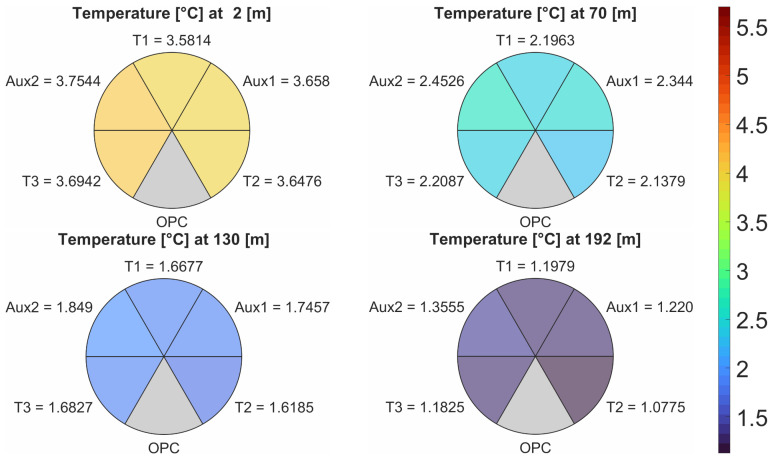
Example of enhanced neighboring sensor analysis showing temperature values for sensors across four stages of a profile to 200 m above ground level.

Figure 5 graphically summarizes this example, illustrating the 1 Hz intra-sensor averaged temperatures at each flight stage for all temperature sensors (PT-100) and the auxiliary temperatures measured by the two relative humidity sensors (HYT 271). The temperature values are organized in the same 60° spatial orientation as depicted in Figure 4 to clearly indicate the adjacent sensors (i.e., neighbors). The colors in each plot follow the adjacent color bar and allow for quick visual evaluation of the entire flight through an animation of the 80 (or more) measurement heights.

In this example profile, the temperatures range from approximately 3.6 °C at the surface to 1.2 °C near 200 m AGL, providing an adequate amount of variation to perform this analysis. The first step in this analysis is determining whether the difference between maximum and minimum temperatures observed for each flight stage is below the accuracy requirement to detect nuanced precipitation phase changes, which in this article is defined as 0.5 °C. In this example, the differences are 0.173, 0.3147, 0.2305, and 0.278 °C, and all flight stages show acceptable differences. This result indicates that airflow through the sensors was consistent and that the sensors are healthy.

For the sake of continuing the analysis, assume a 0.3 °C threshold (the manufacturer’s reported accuracy) and evaluate the 0.3147 °C difference seen in the “developed flight 1” stage (i.e., 70 m) as an example of a flagged discrepancy. In this case, the difference between the maximum and minimum temperatures is produced by a PT-100 temperature sensor (T_2_) and a HYT 271 auxiliary temperature measurement (Aux_2_). As a reminder, the HYT 271 auxiliary temperature measurements are not part of the system’s final reported temperature due to its slower time response. Consequently, this difference only indicates an issue if it persists beyond the sensors’ time response. Evaluating only the three temperature sensors used for the system’s reported value (T_1_, T_2_, and T_3_), the difference between the maximum and minimum temperatures is reduced to 0.0708 °C, well bellow the acceptance threshold.

Considering that T_2_ and Aux_2_ are not adjacent, this discrepancy could indicate a regional airflow imbalance. However, this discrepancy only occurs in one out of the four flight stages, and the sensors involved are of different types. Therefore, this difference is more likely caused by the slower time response of the auxiliary temperature measurements, which is 5 Hz instead of 1 Hz (see Table 1). This conclusion is further corroborated by comparing the temperature difference between Aux_1_ and Aux_2_, 0.1086 °C, which are also non-adjacent.

In addition, correlation coefficients are calculated for sensor pairs based on location (adjacent) and type. These coefficients, shown in Table 2, are calculated based on the data for the entire profile. Within a sensor type, the coefficients were above 0.998; within adjacent sensors (mixing types), coefficients were still above 0.98. These high correlations indicate overall sensor health and airflow consistency, enabling measurement assumptions that allow for multi-sensor averaging and increase the system’s accuracy.

Although similar analyses can be performed manually in other atmospheric observation systems, this design’s redundant and concentric sensor placement gives the analyses presented here an algorithmic characteristic that allows for their automation. The implementation of automated measurement quality control and failure-and-recovery algorithms allows these analyses to be performed on large bodies of data (e.g., for all flights in a multi-day field campaign).

The scalability of the automated measurement quality control and failure-and-recovery procedures which are promoted by this innovative design is a critical feature for any WxUAS with aspirations of broad adoption in operational workflows. Therefore, the design presented here is a meaningful contribution to atmospheric observations, serving as a roadmap for future implementations. Additional examples that further validate this design and the quality of its measurements are provided in Section 6.

## 5. Flight Control Integration

WxUAS comprises weather sensors integrated into a UAV specifically designed for measurement operations, which is characterized as a complete measurement system, accounting for the interactions between the sensors, the aircraft, and the measurement environment. This definition implies that a critical distinction between a vehicle payload and a new measurement system is given by its sensor integration level. Besides mitigating the vehicle’s impact on the measurement, weather sensor integration allows the system to autonomously adapt to the environment, optimizing the data collection through innovative sampling strategies.

Flight control integration can take two forms. The first form is through an external computer managing the flight controller, and the second is via direct wiring into and programming of the flight controller’s microcontroller unit (MCU). Both methods have advantages and disadvantages. This section presents their distinctions as means to justify the implementation design choices in the proposed unmanned aerial weather measurement system for low-altitude winter precipitation environments.

An external processing unit has the advantage of higher computational power. In certain cases, the additional computational capability is a requirement to implement the desired autonomous adaptive behavior, such as vision-based navigation in GPS-denied environments. In this integration method, sensors are wired to the external computer, where their data is stored and processed. In most cases, the external computer is integrated into the flight controller via half-duplex serial communication. Through this communication line, the external processor can receive flight data (e.g., position, attitude, airspeed) and send back simple or complex flight commands. Examples of simple commands are “accelerate”, “climb”, and “yaw 30°”. An example of a complex command is: “go to the next waypoint” (defining coordinates, altitude, and travel speed).

Although the additional computational power allows fast processing of complex algorithms onboard, the half-duplex serial communication can become a limiting factor in the system’s reactiveness. The complementary hardware necessary for this integration method adds to the total system weight and power consumption, impacting flight endurance and resiliency. Some cases of poor integration via external computation also create separate navigation and sensor data files requiring post-flight reconciliation, which can lead to errors in the spatiotemporal results provided by the system.

Given the low computational requirements for the aforementioned sensors, the harshness of the low-altitude winter precipitation environment, and the above-mentioned integration drawbacks, the WxUAS solution proposed in this research uses the direct flight controller integration method. In this method, sensors must be directly wired to the available communication buses, and their processing, storage, and communication software source-codes must be compiled and flashed directly to the flight controller’s MCU.

Although the direct integration method has the benefit of weight, power, and cost reduction, it requires a very steep learning curve for MCU programming. This method also has the added risk of total system failure due to poor programming causing slow processing loops and segmentation faults. To mitigate these issues and enable cross-platform benefits, the proposed WxUAS adopts an open-source vehicle control software framework. The ArduPilot vehicle control framework was created in 2007 and is maintained by nearly 500 developers across the globe. Its source code repository has over 41,000 commits.

**Figure 6:**
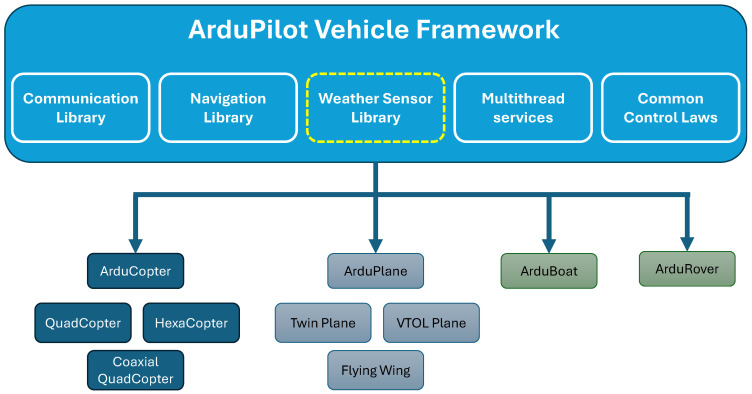
Diagram depicting the ArduPilot open-source vehicle control software framework with this research’s weather sensor integration library. This architectural solution enables the seamless and standardized cross-platform use of the proposed in situ sensor design.

As an autonomous vehicle control framework, ArduPilot encompasses a large variety of vehicles, ranging from aircraft to submarines. Figure 6 illustrates this architectural solution, highlighting how shared features are inherited by each vehicle class and how specific vehicles can be developed as implementations of classes. Through this software architecture, developers can expand the range of vehicle implementations via class extension and create minor vehicle modifications via parametrization of existing vehicles. ArduPilot is freely available to be downloaded and further developed through a GPL3.0 license.

Leveraging the framework’s multi-thread infrastructure that separates the control and service loops, this research’s direct sensor integration solution was implemented as a native library inside the ArduPilot autonomous vehicle framework. As highlighted by the dashed yellow line in Figure 6, each vehicle class also inherits the autonomous vehicle weather sensor library, allowing for seamless and standardized cross-platform use of the in situ sensor package design proposed in this article.

**Figure 7:**
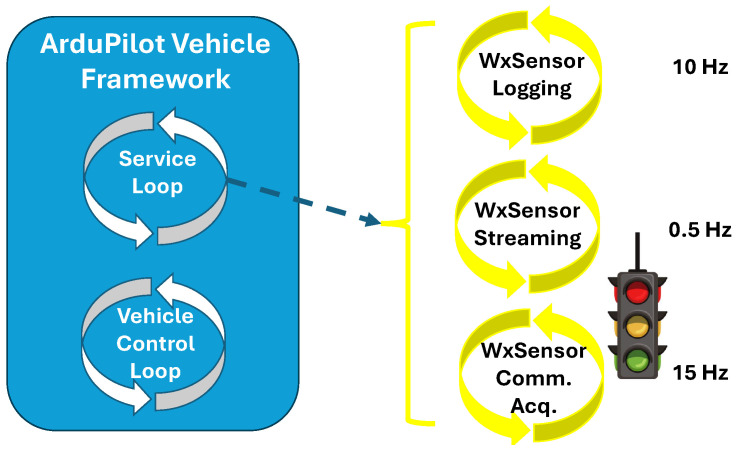
Diagram depicting the weather sensor library’s use of the framework’s service loop as a mitigation strategy for the risks associated with the direct integration method.

As part of the framework, the weather sensor library has direct access to all other framework libraries and services (e.g., navigation, communication, and multithreading). Access to the multithreading services enables the development of software strategies to mitigate the above-mentioned direct integration risks. For example, as illustrated by Figure 7, the weather sensor library can use the framework’s service loop to communicate with the sensors, acquire new data, log the data to the onboard SD card, and stream live data to the ground station computer. Because these tasks are in a separate and lower-priority computational loop, errors and runtime exceptions within these tasks cannot hinder the vehicle’s control and operation.

As also illustrated by Figure 7, each of the tasks mentioned above is performed in asynchronous sub-loops at different rates. This strategy ensures that sensor information is always updated while avoiding costly I/O routines and minimizing the risk of overwhelming the telemetry radio link. Additionally, this strategy divides the failure risks, creating opportunities for data recovery. To implement this strategy, the library submodule responsible for sensor communication and data acquisition uses the SPI and I^2^C buses to request and collect sensor data. At a separate time, the logging submodule uses the framework’s I/O library to save measurements into the flight data SD card. Because each entry in the internal SD card is associated with a time, position, and attitude, the sensor data in the SD card are automatically logged in a spatiotemporal representation. Finally, at a reduced rate, real-time data are transmitted to the ground station computer to provide system users with indications of atmospheric conditions during the flight.

Although the adopted sensors have 1 and 5 s time responses, sensor data are acquired at 15 Hz and logged at 10 Hz. The sensors’ faster oversampling and slightly slower measurement logging (still one order of magnitude faster than the sensors’ time response) ensure that any sensor step change is always captured. Repeated samples are naturally eliminated through the 1 s intra-sensor average mentioned in Section 4. To avoid errors associated with racing conditions, a semaphore is used in the sensor data acquisition submodule. To ensure data integrity, only the sensor data acquisition submodule has access to write on the sensor measurement variables. The remaining submodules have read-only access to the sensor measurements.

**Figure 8:**
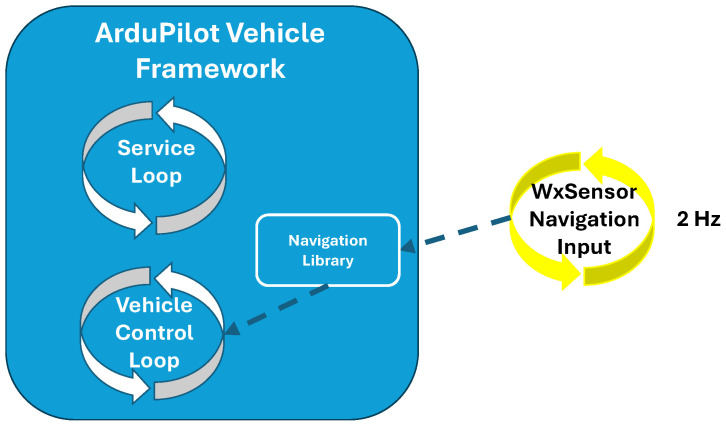
Diagram depicting the weather sensor library producing inputs to the control loop under the safety of the framework’s navigation library.

In addition to eliminating redundant hardware (e.g., the external computer and sensor data streaming link), the direct integration method enables adaptive sampling and other sensor-based autonomous decisions. To implement these features in alignment with the previously discussed direct integration risk mitigation strategies, the weather sensor library leverages its native state in the vehicle framework to safely provide inputs to the vehicle control loop through the framework’s navigation library. This layered implementation through the framework’s architecture separates sensor communication and processing from adjustments to the flight and sampling patterns, ensuring minimum risk to the control loop and providing a safe development environment. Figure 8 illustrates this concept.

**Figure 9:**
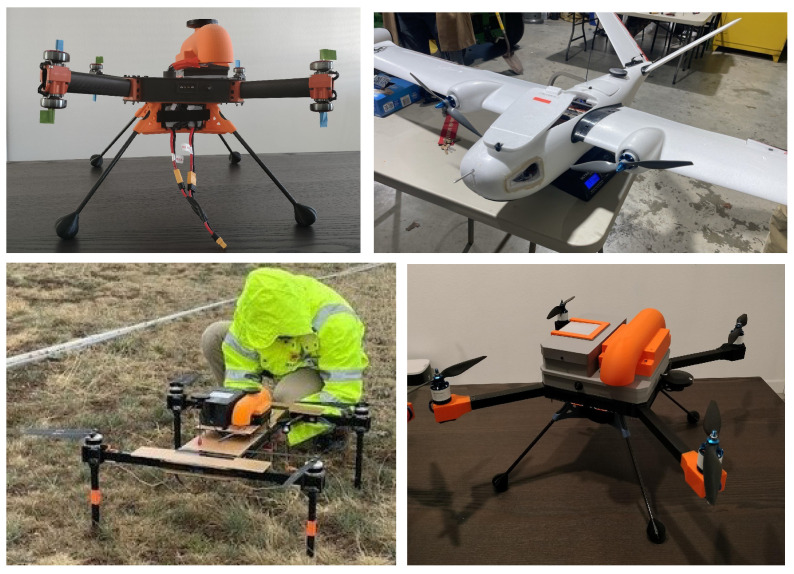
Examples of cross-platform use of the proposed WxUAS in situ sensor hardware and software.

The direct integration of weather sensors into the flight controller through an open-source vehicle control framework as a native library enables the seamless and standardized cross-platform use of the in situ measurement design outlined in this article. Figure 9 presents a few examples of WxUAS in which the proposed design was deployed during this research’s development phase. These examples include a fixed-wing vehicle utilizing the weather sensor library via the ArduPlane class as well as three rotary-wing vehicles incorporating the proposed in situ hardware and software designs. The rotary-wing examples comprise a coaxial quadcopter with eight motors and two different sizes of conventional quadcopters.

## 6. Demonstration

New atmospheric measurement systems must undergo a maturing process that starts with laboratory testing, followed by exploratory research in small-scale investigative field campaigns alongside well-established systems, concluding with an extended improvement and hardening phase in a realistic testbed [24]. This lengthy maturing process is necessary because errors associated with static and dynamic properties, as well as exposure are not present in most laboratory settings. Therefore, operational system characterization requires several years. Within this article’s scope, the first two phases of this maturing process were accomplished through a series of controlled experiments at the Oklahoma Mesonet’s calibration laboratory (Section 6.1), as well as through field deployments with collocated well-established ground-based instruments in Oklahoma (Section 6.2) and Colorado (Section 6.3).

### 6.1. Laboratory Experiments

**Figure 10:**
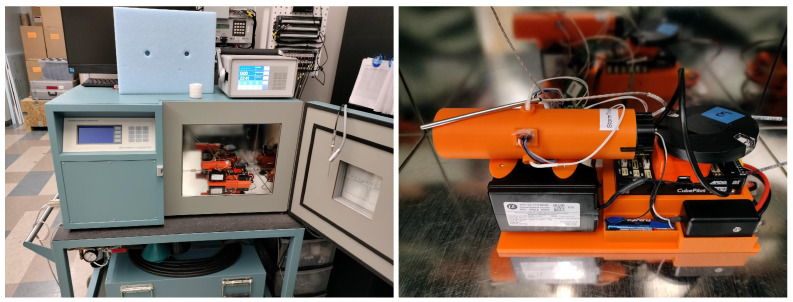
In situ inner shield calibration at the Oklahoma Mesonet’s Calibration Laboratory. The left panel shows the Thunder Scientific 2500 benchtop humidity generator and the right panel details the experimental setup inside the chamber’s test section.

Initial calibrations at the Oklahoma Mesonet’s Calibration Laboratory used the TS 2500 benchtop humidity generator (left panel in Figure 10), manufactured by Thunder Scientific (TS), Albuquerque, NM, USA. This chamber produces controlled temperature and relative humidity conditions following NIST’s two-pressure method. Within this controlled environment, the design’s inner shield was taken through three 3-hour ramps where temperature varied from 5 to 15 °C and relative humidity varied from 65 through 95 %RH. These ranges were selected to represent cold and humid environments within the limitation of this chamber. Although this temperature range does not reflect the operational conditions in freezing precipitation, it still serves as an early developmental evaluation. Additionally, the approximately linear behavior of PT-100 bead thermistors from −15 to 15 °C enables the determination of its calibration coefficients using the above-freezing sub-range. Nonetheless, future work will repeat these experiments in a controlled chamber that supports below-freezing temperatures.

**Figure A2:**
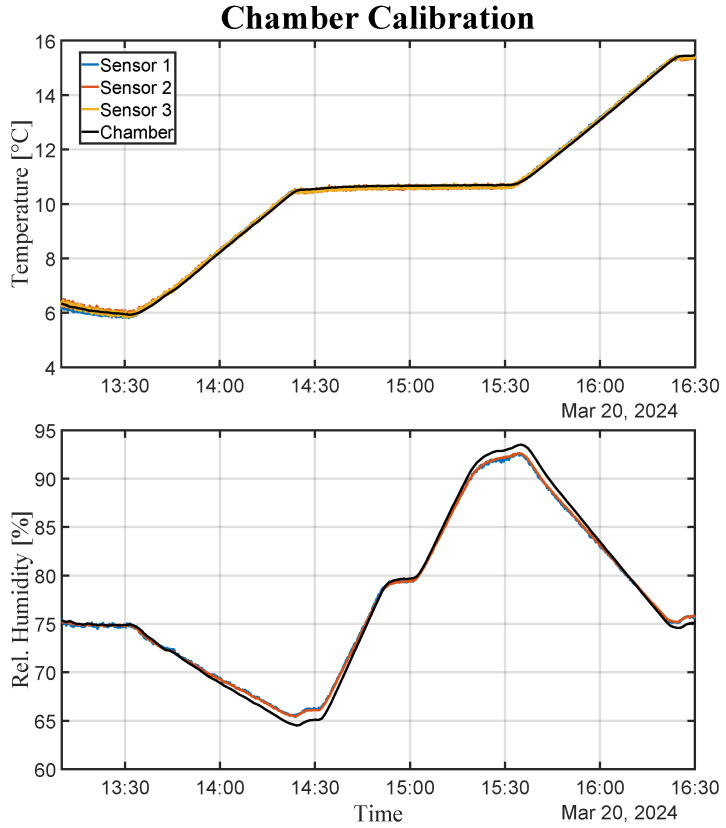
Time series for the temperature and relative humidity experiment at the Oklahoma Mesonet’s Calibration Laboratory. The black series represents the chamber reference values and the colored series represents the calibrated individual sensors.

After determining the calibration coefficients for each sensor, the intra- and inter-sensor averaging procedures (described in Section 4) were applied, and the system’s temperature and relative humidity were calculated. Plots illustrating one of these three experiments are provided in Figure A2. For this example, the mean and maximum absolute errors (MAE and MxAE) for temperature and relative humidity are presented in Table 3.

Although a calibration procedure such as this is expected to improve static errors, the small mean errors indicate the proposed in-situ inner shield design also has a predictable dynamic behavior across different combinations of individual and simultaneous variations in temperature and relative humidity (see Figure A2). This finding is further corroborated by the maximum errors, which are below our self-imposed accuracy standards of 0.5 °C and 2.5 %RH. This result is meaningful because it indicates that in three hours, that is, in 10,800 samples, not one sample crossed the accuracy threshold expected for the mean.

### 6.2. Field Experiment—OKMesonet

**Figure 11:**
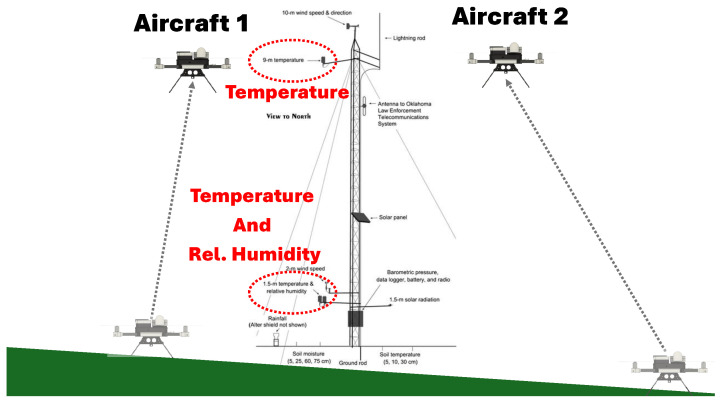
Experimental setup for the system’s performance evaluation under operational conditions. Two identical WxUAS were flown next to the reference Oklahoma Mesonet 10 m tower to obtain collocated measurements.

To evaluate performance under operational conditions, the system was deployed near an Oklahoma Mesonet (OKMesonet) tower for collocated measurements. Each OKMesonet station has a collection of instruments placed at heights that range from 60 cm below to 10 m above the surface. This experiment leveraged the temperature sensors at 1.5 and 9 m and the relative humidity sensor at 1.5 m. Figure 11 offers an illustration of the tower’s sensor placements along with a depiction of the experimental setup.

In this comparison experiment, two identical rotary-wing WxUAS, with the proposed in-situ measurement design solution, were placed on the ground next to the tower (one on each side). After a period of baseline measurements without any effects introduced by the aircraft’s flight characteristics, both aircraft took off and hovered next to the tower at 9 m above the ground (height of the temperature sensor). This hover height was selected to eliminate any potential recirculation produced by aircraft ground effects, which could bias the experiment and interfere with the time series of this operational OKMesonet station. For similar reasons, each aircraft flanked the tower on opposing sides, facing the mild incoming breeze.

Table 4 presents each aircraft’s mean and maximum absolute errors. The temperature errors presented similar values to those obtained in the laboratory experiments. Mean errors were lower than the abovementioned 0.5 °C standard by one order of magnitude, while maximum errors were almost 0.2 degrees below the sensor manufacturer’s reported accuracy. Unfortunately, the same cannot be said about the relative humidity results, which presented mean errors between 6 and 7 %RH and maximum errors above 7.5 %RH. Thankfully, this divergence can be explained by the experimental setup and conditions. Therefore, this result does not indicate poor performance or a design flaw.

As previously discussed, the tower only offers relative humidity measurements at 1.5 m, and the data collection height was set at 9 m to avoid interference from rotor wash recirculation. In many atmospheric conditions, this experimental limitation is deemed acceptable because %RH values at 9 m are assumed to be the same as at 1.5 m, as long as temperatures at both levels are the same. Given the limited availability of relative humidity measurements above 2 m, this comparison limitation is well known and documented in the scientific literature [21,22,25].

To increase the experiment’s representativity for winter weather, data collection was conducted at night when temperatures were near freezing. Unfortunately, the 2024 winter in Oklahoma was atypically warm, with average monthly temperatures at the selected OKMesonet tower during January–March 2024 being 1.5, 10.1, and 13.2 °C. These warm conditions limited the number of near-freezing testing opportunities. Additionally, during most of these opportunities, the atmospheric conditions available produced considerable vertical temperature gradients. As presented in Figure A4, the reported mesonet temperatures during the experiment fluctuated around 4.08 and 5.67 °C at 1.5 and 9 m, producing a 0.21 C m^−1^ gradient.

**Figure A3:**
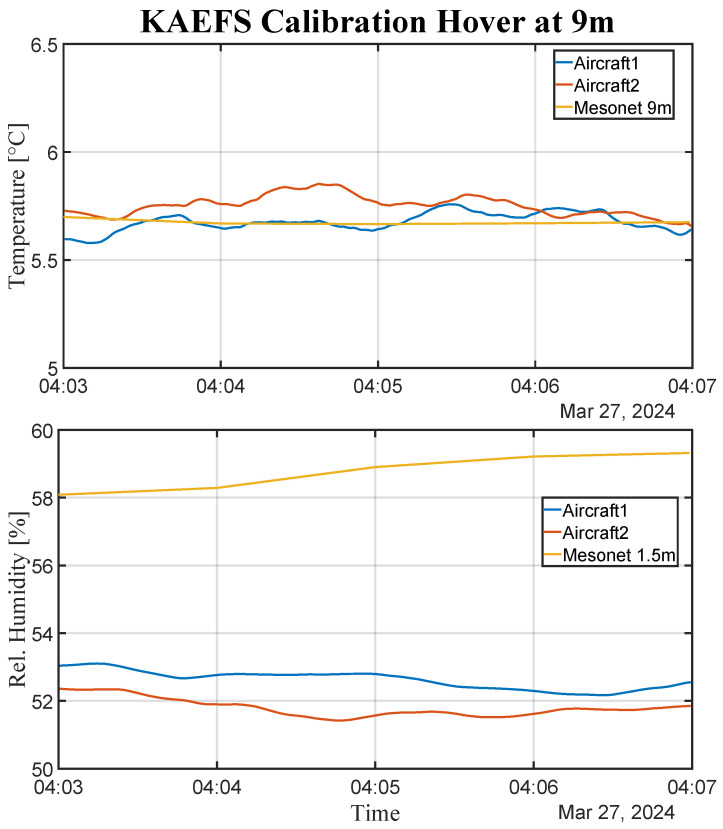
Time series for the tower and two aircraft, highlighting the comparison interval. The mean and maximum absolute errors can be found in Table 4.

**Figure A4:**
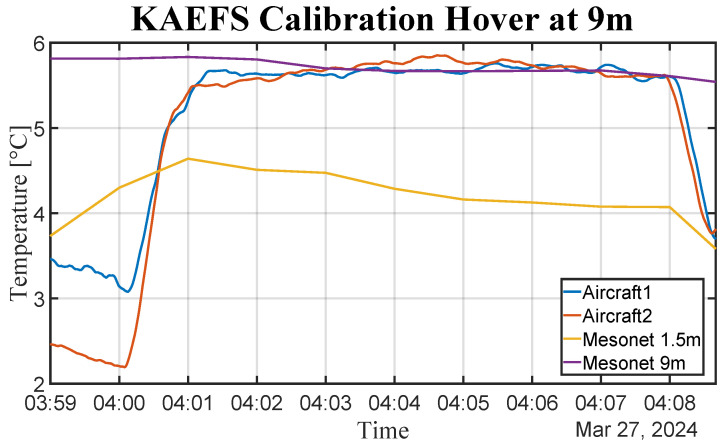
Temperature time series for the tower data (1.5 and 9 m) with data for both aircraft, including data points taken before and after take-off.

**Figure A5:**
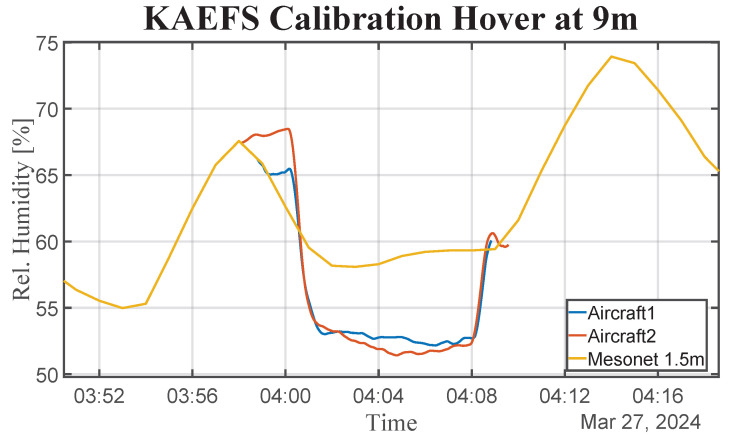
Relative humidity time series for the tower data (1.5 m) with data for both aircraft, including data points taken before and after take-off.

The 0.21 °C m^−1^ gradient found in this experiment’s data invalidates the assumption of humidity homogeneity between 1.5 and 9 m, as indicated by the mean absolute errors between 6 and 7 %RH. Nonetheless, further analyzing the experimental results reveals a few positive insights into the system’s performance for relative humidity measurements in operational conditions. Appendix C provides extended experimental details, and Figure A3, Figure A4 and Figure A5 depict the time series for the resulting data.

The tower data time series in Figure A5 reveals that the tower experienced relative humidity fluctuations larger than 10 %RH before and after the experiment, with a reasonably stable period during the data collection. The same behaviors can be seen in the data for both aircraft, indicating that the differences in absolute values are likely due to the differences in temperatures between 1.5 and 9 m. Additionally, during the stable data collection period, the maximum difference between both aircraft measurements was less than 1 %RH, indicating similar conditions experienced by both systems at 9 m and measurement consistency. Finally, considering the reasonable agreement between both aircraft and the tower in the short period before take-off and after landing, coupled with small errors in temperature measurements, it is reasonable to assume %RH measurements would also have acceptable errors.

Analyzed as a whole, the minor chamber errors for temperature and relative humidity, the minor temperature errors during flight, the relative humidity agreement between aircraft, and their agreement with the tower from the ground indicate the proposed two-layer in situ measurement design does not hinder the sensor’s ability to accurately describe atmospheric conditions. This conclusion is further corroborated by results in Section 6.3, which provides more details on the field deployment at the Research Applications Laboratory’s field station.

### 6.3. Field Experiment—Marshall

Additional demonstration of the WxUAS’s in situ sensor housing design was performed through an intercomparison study conducted at Marshall Field Station, home to the National Science Foundation (NSF) National Center for Atmospheric Research (NCAR) Aviation Applications Program. This field station is located on the outskirts of Boulder (CO), 1627 m above sea level (ASL). It has instrumented towers, a distrometer, and a ceilometer, among other instruments. This study was conducted in coordination with the FAA’s TAIWIN team, which provided the low-altitude precipitation forecast that determined our deployment date during a winter weather event in April 2023.

During this winter weather event, the field station’s disdrometer indicated the presence of drizzle, rain, snow, rain/drizzle, and soft hail at various intensities. The reference tower, with wind velocity, temperature, and relative humidity measurements at 2, 3, and 10 m, indicated winds between 0 and 5 m s^−1^, temperatures between −1 and 2 °C, and relative humidities between 93 and 99 %RH. The field station’s ceilometer, a Vaisala CL31, reported cloud bases as low as 80 m above ground level (AGL). These conditions presented an excellent test opportunity, enabling the initial validation of many system features.

**Figure 12:**
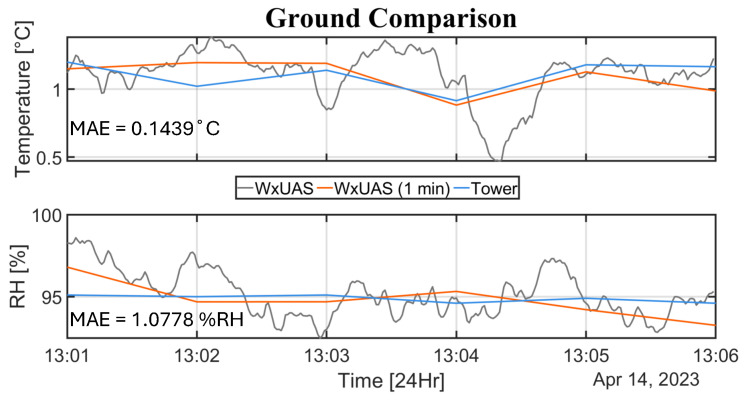
Results for the second temperature and relative humidity ground-based intercomparison experiment. The gray time series represents the WxUAS official reported values at 1-s intervals. The red line represents a 1-min average of the WxUAS data reported at the start of each minute, following the same reporting procedure as the tower data.

The study began with two ground-based experiments to eliminate any potential interference caused by the rotors, creating a control case for the later intercomparison in the Hover flight mode at 10 m (AGL). In these comparison periods, the mean absolute errors were 0.2155 °C and 1.1653 %RH (not shown), and 0.1439 °C and 1.0778 %RH (Figure 12). Both results are below the sensing element accuracies specified by the manufacturers, which are 0.3°C and 1.8 %RH.

During these ground-based experiments, the Marshall Field disdrometer reported snow with total counts increasing from 0 to 400 particles per minute. During this same period, the 77 GHz vertically pointing radar (part of the remote sampling design proposed in this research) detected a 15 to 25 dB increase in reflectivity, with Doppler velocities of −2 and −4 m $s^{-1}$. In addition, the optical particle counter inside the two-layer in situ sensor shield did not report any change in particle size distribution during this period.

**Figure 13:**
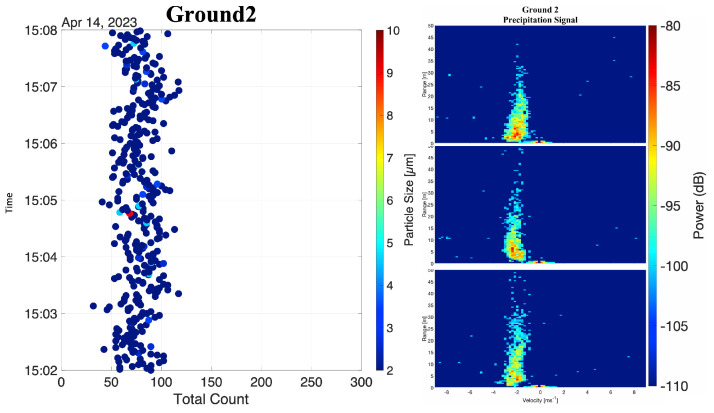
Results for particle size distribution and range-Doppler map during the second ground-based experiment. For the vertically pointing radar, negative Doppler velocities indicate particles falling towards the radar.

These results are shown in Figure 13, where the left panel presents the temporal evolution of the particle size distribution’s mode and the right panel presents three range-Doppler maps. Evaluating the particle size distribution’s mode, it is possible to note that the distribution’s bin with the highest count remained between 0 and 2 μm, with a total count between 50 to 100 particles per second. These rates and distribution modes are consistent with the sensor’s background readings.

The results from these ground experiments reveal that, during active precipitation, the proposed in-situ measurement design effectively utilized gravity to mechanically separate large precipitation from the air, avoiding impinging the system’s sensing elements. Additionally, the small mean absolute errors in temperature and relative humidity indicate the design strategy does not introduce any bias or artifacts. Combined, these results help validate the proposed design and confirm our expectations regarding the mechanical separation of large precipitation drops from the air while eliminating the impact of radiative and sensible heat fluxes.

### Cloud Droplet Size Distribution Profiles

Given the novelty of WxUAS cloud droplet size distribution profiles, methods for validating the proposed design are not yet well established. Additionally, considering the physical complexity of clouds, controlled chambers with artificially generated clouds are not abundantly available. Therefore, the initial tests for the proposed WxUAS in situ particle size distribution measurements in the 2 to 40 μm range were executed at the Marshall Field Station using naturally occurring conditions.

Leveraging the atmospheric conditions during the previously mentioned winter weather event, four vertical profiles from the ground to 120 m were performed to evaluate the WxUAS’s ability to characterize the atmosphere’s thermodynamic structure and its microphysical properties in near-freezing conditions. Although the WxUAS was designed to perform profiles up to 1500 m, flight ceilings were limited for compliance with the FAA’s Part 107 rules. This limitation was unfortunate, as it could have hindered the system’s ability to observe any meaningful structural change with height. However, given the field station’s proximity to the mountains, its elevation (1627 m, ASL), and the intensity of the weather event, the four profiles were performed in scientifically relevant conditions that permitted gauging the WxUAS’s potential.

**Figure 14:**
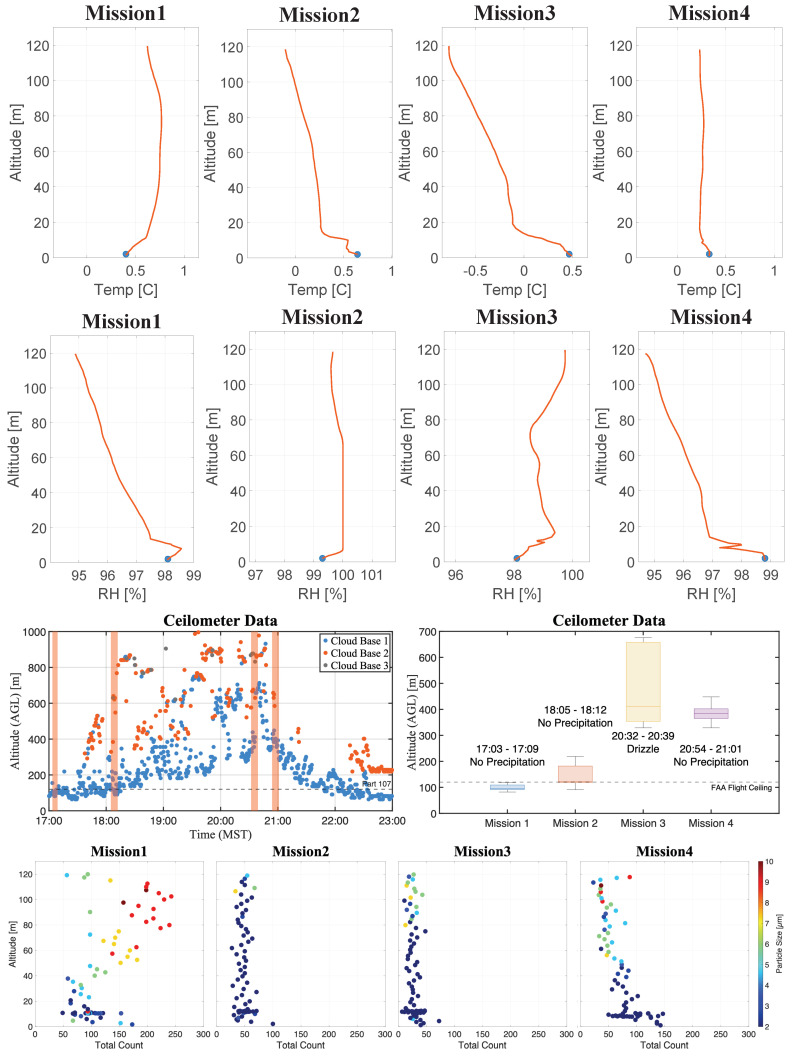
Results for four atmospheric profiles from the surface to 120 m: (**Top**) temperature and relative humidity, (**Center**) ceilometer data indicating the cloud base, and (**Bottom**) cloud droplet size distribution profiles. The orange-shaded columns in the ceilometer’s scatter plot represent the time intervals for the four flights.

The results from these profiles are presented in Figure 14. In it, the top panels depict the WxUAS temperature and relative humidity profiles (indicated by red lines) juxtaposed with the tower’s sensors at 2 m (represented by blue dots), while the center panels display the ground-based ceilometer data against the cloud droplet size distribution profiles (bottom panels). In all plots, the *y*-axis signifies the altitude above ground and the *x*-axis corresponds to the variable of interest. For the cloud droplet size distribution profiles, the data represent the distribution’s mode at each altitude, that is, the cloud droplet size bin with the highest count. The color in the profiles denotes the mode’s droplet diameter, while the *x*-axis portrays the distribution’s total count for that altitude. For the ceilometer scatter plot, the orange shaded columns represent the time intervals for the four flights.

The first profile occurred immediately after an intense snow event had subsided, while the ceilometer reported a low-altitude cloud base near 95 m AGL (Figure 14). The snow, freshly deposited on the ground, produced lower temperatures and intense moisture on the surface layer, as reported by the tower instruments. These conditions were also reflected in the WxUAS measurements, most notably in the optical particle counter data, which showed cloud droplet size distribution modes with total counts above 150 particles per second on the surface, falling to 100 between 10 and 50 m AGL and rising to 200 between 80 to 110 m (Figure 14: ‘Mission 1’).

The high count of smaller particles (from 0 to 4 μm) near the ground is most likely produced by the rotor wash recirculation during takeoff over the snow-covered surface. While other effects could yield similar increases in small particle count, the likelihood of rotor wash effects is substantiated by the similar pattern observed during ‘Mission 4’ following a drizzle event. Additionally, this pattern is notably absent in ‘Mission 2’ and ‘Mission 3’ after periods without precipitation and during the ground-based comparison experiments (Figure 13), which were conducted during a snowfall event with the rotors turned off.

The high count of larger particles (i.e., cloud droplets from 8 to 13 μm) from 80 to 110 m shows agreement with the ceilometer’s reported cloud base, which had a median altitude of 95.5 m, interquartile range (IQR) of 18 m (between 91 and 109 m), and 82 to 118 m minimum and maximum values. This result demonstrates the system’s ability to detect the smaller cloud particles and characterize the cloud layer’s depth.

Furthermore, upon comparing the four cloud droplet size distribution profiles with the ceilometer reports, the WxUAS’s high sensitivity to the smaller cloud droplets becomes evident. During the interval between ‘Mission 1’ and ‘Mission 3’, as the reported cloud base ascended from 95 to 400 m and subsequently descended in ‘Mission 4’ and beyond, the distribution mode profiles identified by the WxUAS began to manifest the size variation with altitude consistent with the ceilometer reports, albeit earlier. For instance, during ‘Mission 3’, the reported cloud base exhibited a median altitude of 411 m with an IQR of 305 m. Concurrently, the cloud droplet size distribution profile reveals an increase in particle sizes above 80 m.

During ‘Mission 4’, the reported cloud base is characterized by a median altitude of 384 m and an IQR of 37 m, corresponding to an increase in particle sizes above 60 m, with particles reaching 11 μm at 120 m. Approximately 40 min after ‘Mission 4’, the ceilometer reported the cloud base at 137 m with an IQR of 91 m. One hour later, the ceilometer recorded the cloud base at 82 m with a 27 m IQR between 73 and 100 m.

The above-mentioned results indicate the system properly shielded its sensing elements from precipitation while accurately describing low-altitude atmospheric conditions with enhanced sensitivity in its operational environment. Although these results reflect the conditions of only one winter weather event, this event presented considerable variability and exposed the system to dry conditions, clouds, drizzle, rain, snow, rain/drizzle, and soft hail. In conjunction with the results from the intercomparison study conducted in Oklahoma and the controlled chamber results, this operational environment experiment provides initial validation of the proposed WxUAS in situ measurement system design.

## 7. Discussion

As pointed out in this article’s introduction, there is a need for high spatiotemporal resolution weather data in the atmospheric boundary layer to support current and future aviation operations, as well as other societal weather-dependent activities (e.g., agriculture and power distribution). Besides motivating the development of the new measurement method presented in this article, the lack of weather data in the lower atmosphere for direct measurement comparison also creates a considerable challenge for validating new measurement technologies. For this reason, this article followed a multi-stage and multi-point approach, comprised of a partial system test in controlled environments (i.e., test chambers), a complete system intercomparison in semi-operational conditions, and a multi-instrument intercomparison in operational conditions.

Although under different conditions, each stage provides pertinent information for validation of the critical system features. In conjunction, these stages increase confidence in the previously drawn conclusions. Nonetheless, it is also important to discuss the limitations of this method, particularly regarding the limitations of the inferences drawn from the results presented here, as well as the future developments needed for the advancement of this research field.

The lack of a direct comparison source for validation of WxUAS is a multidimensional issue. Part of this issue is associated with the WxUAS mobility, which gives it an operational range in the scale of a few kilometers. This operational range implies that comparisons with other in situ sampling solutions are limited to a small number of samples. For example, comparisons with towers can produce only one or two comparison points, while comparisons with radiosondes often produce at most 15 comparison points in cases with active drift compensation [20,26]. This limitation occurs because towers are fixed in space, and radiosondes have high ascent rates through the boundary layer due to the amount of helium necessary to make them reach higher atmospheric layers.

To mitigate this issue and increase the opportunities for comparison with fixed instruments, the method used in this study leverages additional experiments outside the system’s final operational conditions (e.g., ground and hover comparisons). Although these additional experiments provided limited information regarding the system’s operational performance, their results can be used to validate subsystem features, and the joint analyses of their results can validate complex operational features. For example, a hover experiment cannot provide insight into the WxUAS’s profiling performance. However, the absence of an increase in mean and maximum measurement errors relative to the reference ground-based measurement when comparing results from the ground experiment (propellers off) to the hover experiment (propellers on) indicates the strong likelihood that the profiling measurements will not suffer from propeller induced bias. This inference is possible because the theory of gust response in flight dynamics indicates that a vehicle has a better response to external disturbances in higher thrust-to-weight conditions [27]. In other words, in a profile flight (ascent) a UAV is more stable and produces fewer propeller variations than in hover. Additionally, during a profile, a WxUAS has a continuous source of undisturbed incoming flow, while in a hover, the WxUAS is more prone to measurement errors due to recirculation [20]. However, the use of these additional experiments is only valid if all experiments present similar error characteristics.

Another dimension of the lack of direct comparability within comparisons with other in situ atmospheric measurement systems is the difference in measurement scales. For example, even in collocated comparisons where sensors measure the same atmospheric variable (e.g., temperature), the compared values are often measured and reported in different time scales. This time scale difference is due to different sensor time responses, the use of physical filters, or the employment of longer time scale averaging to increase system accuracy [20,28]. This issue is exemplified in this article by the ground-based experiment at Marshall (shown in Figure 12). In this example, both issues are present. The tower employs a 1-min averaging technique and has a sensor time response in the scale of minutes, while the WxUAS reports a new measurement every second, with a time response slightly below 1 s. This discrepancy allows the WxUAS to capture much finer fluctuations in atmospheric temperature. However, without a direct fast-response reference, the presence of fluctuations cannot be asserted because system noise could also yield similar behaviors. Therefore, the successful comparison of the averaged WxUAS fluctuations to the 1-minute reference data only serves as an indication of overall accuracy, and not as definitive proof of precision and sensitivity.

An alternative to addressing the WxUAS’s mobility and extended range is to compare its measurements with remote-sensing instruments. However, this type of comparison suffers from the inference limitations of differing measurement technologies. For example, in the evaluation of the WxUAS’s ability to detect and locate cloud layers, the WxUAS cloud droplet size distribution profiles were compared to the collocated ceilometer’s cloud-base height. However, because these two systems use different technologies to measure slightly different atmospheric characteristics, their comparison is based on the inferences made from their measurands (i.e., light scattering into cloud-base height and light-based discrete particle counting into cloud droplet size distribution). Therefore, these comparisons carry the measurement inference errors of each system.

Considering the novelty of WxUAS measurements, these limitations are still a part of its current state-of-the-art research, as reflected by the related literature [14,17,20,21,22,25,26,29,30,31]. Therefore, the development of these technologies creates a need for calibration and validation facilities with the capability to emulate the operational conditions for at least pressure, temperature, humidity, and wind on a physical scale where the full system can be validated. Such facilities should also be capable of supporting the added complexity of flight dynamics. Given the magnitude of this challenge and its unlike achievement in the short term, other fast-response comparison methods should be devised to improve compound methodologies such as the one described in this article. Among the recommended intermediary improvements, we highlight arrays of instrumented tall towers and tethered lifted systems. These are considered intermediary solutions because although they improve the number of comparison points and the availability of similar technology measurements, they still rely on naturally occurring variations in atmospheric conditions.

### Operational Challenges

This article has introduced a novel unmanned aerial weather measurement system designed for sampling the low-altitude freezing precipitation environment. Consequently, it is important to also present at least a brief discussion on the operational aspects of WxUAS measurements, especially concerning the challenges posed by near-freezing temperatures.

Rotor wash—Although UAV-based sampling systems leverage their mobility for high-resolution atmospheric measurements, they can also present some limitations near the surface. For example, the layer mixing induced by rotor wash in multi-rotor vehicles can produce data artifacts below 10 m, as shown by the particle size distributions for ‘Mission 1’ and ‘Mission 4’. In both cases, precipitation on the ground seemed to induce a higher particle count. Therefore, in cases where features near the ground are important, vehicles should be designed for low rotor wash and their limitations should be properly characterized. Accommodating use in different vehicles and modified applications is not an issue for the technology presented in this article because it was developed via integration into the flight controller. Besides eliminating redundancies in the sampling system, this integration makes the presented solution platform-agnostic and multimodal (i.e., for fixed or rotary-wing UAVs).

Sensor equilibrium—Some UAS components require additional care in near-freezing environments (e.g., the flight controller, batteries, and propellers). For this reason, keeping the system in a heated environment between flights may be necessary. Unfortunately, keeping the aircraft warm also means warming the sensors. Therefore, allowing the sensors to reach equilibrium with the environment is essential before starting a new profile. The time required for sensors to reach equilibrium with the sampling environment depends on the sensor aspiration rate and the total system time response. For the data presented in this article, given the slowest sensor’s (i.e., HYT 271) 5 s time response (i.e., t_63_), the 3 min necessary for pre-flight checks provided more than enough for proper sensor equilibrium to be reached.

Low-altitude forecasting—Our field deployment was strategically guided by the forecast expertise of the FAA’s TAIWIN team, complemented by the support of meteorologists from NCAR’s Aviation Applications Program. Their precise forecasting efforts positioned us amid a winter weather event characterized by various precipitation types, enabling testing and validation of the WxUAS. However, it is crucial to highlight that even these experts encountered limitations in the available tools for providing low-altitude hourly forecasts to guide our flights. Notably, in the case of ‘Mission 3’, the preparation to fly and sample drizzle was triggered by a human observer who reported the incoming weather from a strategic vantage point. These limitations further highlight the importance of novel technology to support their work.

Flight restrictions—Low-altitude freezing precipitation poses a significant yet largely undetected and unforecasted hazard for manned and unmanned aircraft. To conduct an effective study of this environment that accounts for the precipitation phase changes between the generation and the surface layers, comprehensive sampling across the entire airspace between them is essential. Achieving this may require the systems to sample from the surface to 2000 m, potentially even penetrating cloud layers. However, existing regulations imposed by the FAA restrict these operations for UAS due to the associated risks.

For this intercomparison study, we partnered with the Rocky Mountain Metropolitan Airport (KBJC) and Vigilant Aerospace to submit an altitude and cloud restriction waiver request. In our proposed setup, we would fly up to 600 m inside the KBJC’s class D airspace (under tower control) and would deploy Vigilant Aerospace’s Flight Horizon System as a redundant electronic detect-and-avoid system. The Flight Horizon System employs a ground-based radar and ADS-B receiver to project potential collision trajectories for cooperative and non-cooperative aircraft, and provides the Remote Pilot-In-Command (RPIC) with an avoidance maneuver prescription.

Considering the nature of winter weather research, our flights were planned to occur in conditions typically outside the operating capabilities of most non-commercial aircraft, thereby minimizing the risk of airspace conflict. Regrettably, despite our efforts to design a low-risk experimental setup, the FAA did not deem it safe enough, leading to the denial of our waiver request. Nonetheless, the need for this type of research and the characterization of low-altitude freezing precipitation remains. Therefore, in the absence of a regulatory path for this type of research, the safe sustainment of current operations and the introduction of the next generation of low-altitude aircraft (e.g., small Unmanned Aerial Vehicles and Advanced Air Mobility aircraft) in the National Airspace System will likely remain difficult.

## 8. Conclusions

This article offers an initial demonstration of a WxUAS in situ sampling method for low-altitude winter precipitation environments. The data presented herein reflects an early developmental stage, and are meant to create an avenue for early feedback from the winter weather and aviation weather communities. Nevertheless, the provided results showcase key features. Most notably, the sensor shield design exhibits the capability to utilize gravity for the mechanical separation of large precipitation from the air. This functionality prevents the impingement of the system’s sensing elements while ensuring the reduction of bias or artifacts in the data. The mean absolute errors for temperature and relative humidity within the shield are as low as 0.1 °C and 1.1 %RH. Additionally, the cloud droplet size distribution profiles demonstrated the system’s high sensitivity, potentially detecting distinctions between cloud and cloud-transition layers.

Besides illustrating WxUAS’s design capabilities to support low-altitude winter precipitation research and provide novel insight into the mechanisms that govern it, the design concepts for WxUAS-based in situ sampling presented in this article also demonstrate the method’s versatility and scalability. Through its direct integration into the flight controller via a native library within a ubiquitous flight control framework, this WxUAS in situ sampling method becomes inherently cross-platform. Its redundant and concentric sensor placement allows the implementation of automated measurement quality control and failure-and-recovery algorithms, enabling the automated sanitization of large datasets (e.g., for multiple flights in a multi-day field campaign or multiple units of this WxUAS at multiple locations).

Future work on the presented WxUAS will include continued validation against well-established instruments, maturation in adverse operational conditions, and a new multimodal sensor data fusion algorithm for WxUAS-based automated present weather inference. Details on these developments and the current remote sampling method (i.e., WxUAS-based 77 GHz vertically-pointing precipitation radar) will be expounded upon in a complementary article.

## Figures and Tables

**Table 1:** In situ sensor specifications.

	MS5611	PT-100	HYT 271		OPC-N3
Range	1 to 120 kPa	−90 to 50 °C	0 to 100%RH	−40 to 125 °C	0.35 to 40 μm
Accuracy	±1.5 kPa	±0.3 °C	±1.8%RH	±0.2 °C	–
Time resp. (t_63_)	1.7 ms	1 s	1 s	5 s	1 s
Current [ μA]	1	150	850		180 mA
Dimensions [mm]	5 × 3 × 1	30 × 15 × 3	10.2 × 5.1 × 1.8		75 × 64 × 60

**Table 2:** Correlation coefficients for the five sensors in the manual neighboring sensor analysis.

Sensors	T1	Aux1	T2	T3	Aux2
T1	–	–	–	–	–
Aux1	0.9848	–	–	–	–
T2	0.9983	0.9834	–	–	–
T3	0.9979	–	0.9989	–	–
Aux2	0.9870	0.9984	–	0.9809	–

**Table 3:** Mean and maximum absolute errors during the experiment at the Oklahoma Mesonet’s Calibration Laboratory.

Temperature		Relative Humidity	
**MAE**	**MxAE**	**MAE**	**MxAE**
0.0537 °C	0.4856 °C	0.1458 %RH	1.3073 %RH

**Table 4:** Mean and maximum absolute errors during the Oklahoma Mesonet’s flight comparison experiment.

	Temperature		Relative Humidity	
	**MAE**	**MxAE**	**MAE**	**MxAE**
Aircraft 1	0.0361 °C	0.116 °C	6.1565 %RH	7.093 %RH
Aircraft 2	0.0808 °C	0.1856 °C	6.9813 %RH	7.6312 %RH

## Data Availability

Data presented in this article are available at https://zenodo.org/records/14619052 as of 9 January 2025. For more information, please contact the corresponding author.

## Abbreviations

The following abbreviations are used in this manuscript:

AGL	Above Ground Level
ASL	Above Sea Level
AAM	Advanced Air Mobility
IQR	Interquartile Range
HEMS	Helicopter Emergency Medical Services
mmWave	Millimeter-Wave
MRMS	Multi-Radar Multi-Sensor System
NAS	National Airspace System
NCAR	National Center for Atmospheric Research
NSF	National Science Foundation
OPC	Optical Particle Counter
SLD	Supercooled Liquid Droplets
SLW	Supercooled Liquid Water
SWaP	Size, Weight, and Power
TAIWIN	Terminal Area Icing Weather Information for NextGen
UAVs	Unmanned Aerial Vehicles
WxUAS	Unmanned Aerial Weather Measurement Systems

## Appendix A

This appendix offers an illustration of each sensor used in the WxUAS in situ measurement solution (see Figure A1). In addition to the specifications found in Table 1, a frozen version of the data sheets representing their characteristics as of this writing is available in this article’s data repository (see Data Availability Statement).

## Appendix B

Initial calibrations of the sensor inner shield for the in situ measurement design were performed at the Oklahoma Mesonet’s Calibration Laboratory. This procedure used the Thunder Scientific 2500 benchtop humidity generator (left panel in Figure 10). This chamber produces controlled temperature and relative humidity conditions following NIST’s two-pressure method. Within this controlled environment, the design’s inner shield was taken through three 3-h ramps where temperature varied from 5 to 15 °C and relative humidity ranged from 65 to 95 %RH, jointly. As can be seen, the dwell and step periods for each variable were mismatched to produce several test conditions.

## Appendix C

To evaluate whether integration into the aircraft introduced new measurement errors, the system was deployed near an Oklahoma Mesonet (OKMesonet) tower for collocated measurements. Each OKMesonet station has a collection of instruments placed at heights that range from 60 cm below to 10 m above the surface. This experiment leveraged the temperature sensors at 1.5 and 9 m and the relative humidity sensor at 1.5 m. Figure 11 offers an illustration of the tower’s sensor placements along with a depiction of the experimental setup.

In this comparison experiment, two identical rotary-wing WxUAS with the proposed in situ measurement design solution were placed on the ground next to the tower, one on each side. After a period of baseline measurements without any effects introduced by the aircraft’s flight characteristics, both aircraft took off and hovered next to the tower at 9 m above the ground (the height of the temperature sensor). This hover height was selected to eliminate any potential recirculation produced by aircraft ground effects which could bias the experiment and interfere with the time series of this operational OKMesonet station. For similar reasons, each aircraft flanked the tower on opposing sides, facing the mild incoming breeze. The measurements were taken at the Washington site of the OKMesonet, located at the Kessler Atmospheric and Environmental Field Station (KAEFS) in central Oklahoma.
